# Surface Modification of Titanium with BMP-2/GDF-5 by a Heparin Linker and Its Efficacy as a Dental Implant

**DOI:** 10.3390/ijms18010229

**Published:** 2017-01-23

**Authors:** Dae Hyeok Yang, Sang Woong Moon, Deok-Won Lee

**Affiliations:** 1 Institute of Cell and Tissue Engineering, College of Medicine, The Catholic University of Korea, Seoul 06591, Korea; yangdh@catholic.ac.kr; 2 Department of Ophthalmology, College of Medicine, Kyung Hee University, Seoul 02454, Korea; msw@khnmc.or.kr; 3 Department of Oral & Maxillofacial Surgery, Kyung Hee University Dental Hospital at Gangdong, Kyung Hee University, #892 Dongnam-ro, Gangdong-gu, Seoul 05278, Korea

**Keywords:** human bone morphogenetic protein-2, human growth and differentiation factor-5, heparin, bone formation, osseointegration

## Abstract

In this study, we prepared human bone morphogenetic protein-2 (hBMP-2)/human growth and differentiation factor-5 (hGDF-5)-coated titanium (Ti) disc and screw types for controlled release of the growth factors (GFs). The two growth factors were coated onto Ti with a smooth surface using their specific interaction with heparin, because they have heparin binding sites in their molecular structures. Efficacy of the two growth factor-coated Ti for enhancement of bone formation and osseointegration was compared to pristine Ti, and hBMP-2- and hGDF-5-coated Ti in vivo. The surface chemical composition, surface morphology, and wettability characteristics of the metal samples were determined by X-ray photoelectron spectroscopy (XPS), scanning electron microscopy (SEM), and contact angle measurement, respectively. The initial burst of hBMP-2, hGDF-5, and their combination, occurred within one day of the release study, resulting in 12.5%, 4.5%, and 13.5%/3.2%, and then there was a sustained, even release of these two growth factors from the coated metal for 30 days. In vitro tests revealed that MC3T3-E1 cells cultured on the two growth factor-coated Ti had a higher proliferation rate and a higher activity for alkaline phosphatase (ALP), which led to a larger amount of calcium deposition and larger expressions of type I collagen (*COL 1*), *ALP*, and osteocalcin (*OCN*) mRNAs. In vivo animal tests using ten white New Zealand rabbits showed that the two growth factor-coated Ti enhanced bone formation and osseointegration at the interface between the implants and host bone. In addition, histological evaluation showed that bone remodeling, including bone formation by osteoblasts and bone resorption by osteoclasts, actively occurred between the two growth factor-coated Ti and host bone. Consequently, it is suggested that Ti surface modification with the combination of hBMP-2 and hGDF-5 for the two growth factor-coated Ti implants can improve the clinical properties of implants for orthopedic and dental applications.

## 1. Introduction

Titanium (Ti) and its alloys are widely used as implants in dental and orthopedic fields [1]. The successful implantation of such implants depends on their initial osseointegration and long-term permanence in function [1]. So far, various surface modifications, such as laser irradiation, acid-etching, and incorporation of ceramics, have been proposed for the achievement of such purposes [2,3,4]. Nevertheless, the various surface modifications have insufficient for achieving these aims.

Recently, surface modifications with the bone morphogenetic protein (BMP) family of growth factors are being introduced for satisfactory initial osseointegration [3,5,6]. Among these, bone morphogenetic protein-2 (BMP-2) and growth and differentiation factor-5 (GDF-5) have received significant attention from researchers due to their abilities for enhancement of osseointegration and bone formation in vivo [5,6]. In particular, GDF-5 is less known as a growth factor for the enhancement of bone formation, as compared to BMP-2. Some studies proved that GDF-5 plays a leading role in regenerating the bone on damaged periodontal ligament tissue [7,8,9].

However, these biomolecules have short half-lives due to degradation by enzymes, leading to insufficient bone formation. Local delivery of excess amounts of these biomolecules is not desirable as they may induce malignancy [3]. In order to overcome these drawbacks, several additional surface modifications, such coating with hydrogels, click chemistry, and the treatment of 3,4-dihydroxyl-l-phenylalanine (DOPA), have been designed [3,5,10,11,12,13,14].

Heparin is a highly-sulfated glycosaminoglycan and linear natural polysaccharide [15]. It has a binding affinity with various growth factors including BMP-2 and GDF-5 [15]. Surface modification of Ti with heparin enables increasing the half-life of these growth factors [3,5]. In addition, the surface modification may suppress the induction of malignancy by controlling their releases in a sustained manner. There have been some reports about the evaluation of bone formation and osseointegration by BMP-2- or GDF-5-coated Ti [3,5,6,12]; however, their combination effect on the enhancement of bone formation and osseointegration remains unclear. Therefore, this research is, to our knowledge, the first report that confirms the feasibility of dual growth factor-coated Ti on the enhancement of bone formation and osseointegration.

In the present study, we designed and prepared a double-layered human BMP-2/human GDF-5-coated Ti with heparin (hBMP-2/hGDF-5/Ti), and its efficacy on the enhancement of bone formation and osseointegration was compared to pristine Ti, hBMP-2/Ti, and hGDF-5/Ti in vivo. Alkaline phosphatase (ALP) activity, calcium deposition and bone-related mRNA expression including type I collagen (*COL 1*), *ALP*, and osteocalcin (*OCN*) were tested in vitro. Bone volume (BV) and bone volume/tissue volume ratio (BV/TV), bone-implant contact (BIC), removal torque, and histological evaluations were carried out for the in vivo tests.

## 2. Results

### 2.1. Surface Chemical Composition

**Figure 1:**
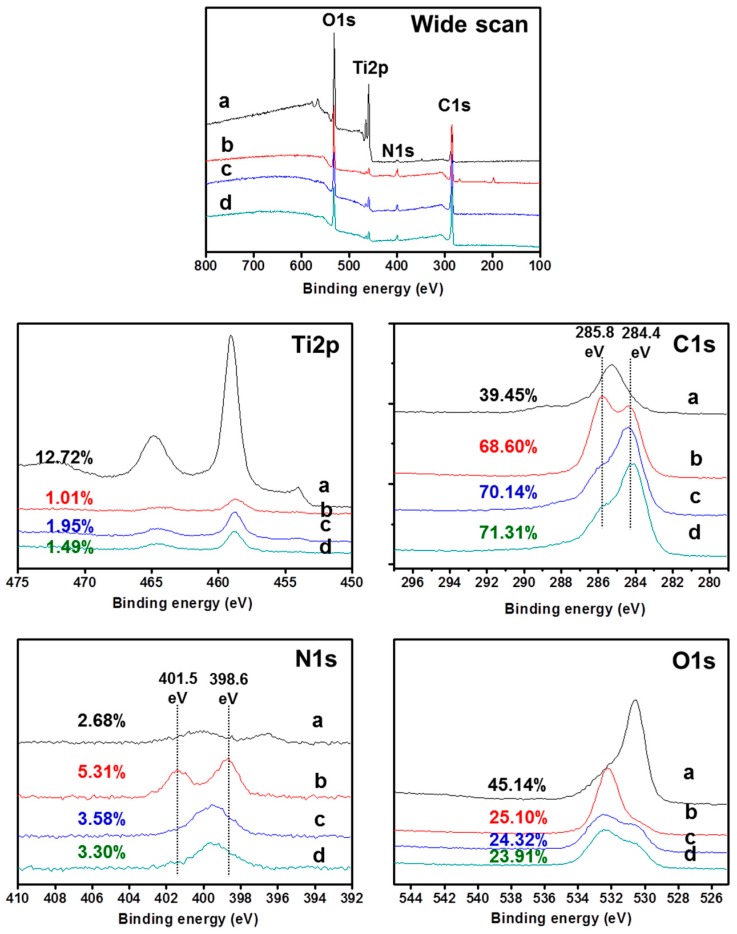
XPS spectra of (**a**) pristine Ti; (**b**) hBMP-2/Ti; (**c**) hGDF-5/Ti; and (**d**) hBMP-2/hGDF-5/Ti samples, as monitored from 100 to 800 eV.

Figure 1 shows the surface chemical composition of pristine Ti, hBMP-2/Ti, hGDF-5/Ti, and hBMP-2/hGDF-5/Ti. Ti2p and O1s XPS signals were observed on pristine Ti. The O1s was attributed to the TiO_2_ layer of pristine Ti. A distinct N1s XPS signal was observed on hBMP-2/Ti, hGDF-5/Ti, and hBMP-2/hGDF-5/Ti, indicating the success of the surface modifications. The surface modifications were further explained by the examination of atomic Ti2p, C1s and O1s percentages. hBMP-2/Ti, hGDF-5/Ti, and hBMP-2/hGDF-5/Ti had lower percentages of Ti2p (1.01%, 1.95% and 1.49%) than pristine Ti (12.72%). Furthermore, the surface-modified Ti samples showed an increased percentage of C1s and a decreased percentage of O1s, as compared to pristine Ti. In particular, hBMP-2/hGDF-5/Ti showed a higher percentage of C1s and a lower percentage of O1s than hBMP-2/Ti and hGDF-5/Ti. These can be ascribed by the surface modification of Ti with hBMP-2, hGDF-5, and their combination, as the number of carbons with attached hBMP-2 and hGDF-5 is larger than that for oxygen for pristine Ti. Furthermore, changes in the high-resolution narrow C1s and O1s spectra clearly supported that the surfaces of Ti were successfully modified with hBMP-2, hGDF-5, or the combination. The surface-modified Ti samples showed two narrow signals in their C1s and O1s XPS spectra, as compared to pristine Ti. The binding energies at 284.4 and 285.83 eV were assigned to –C–C– and –C–O–C–, respectively. The binding energies at 398.6 and 401.5 eV were assigned to –C–NH_2_– and –O=C–NH–, respectively.

### 2.2. Surface Morphology

**Figure 2:**
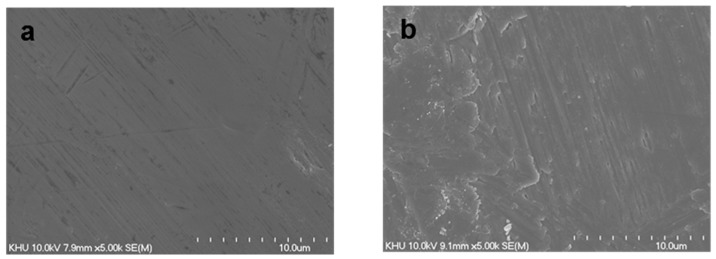

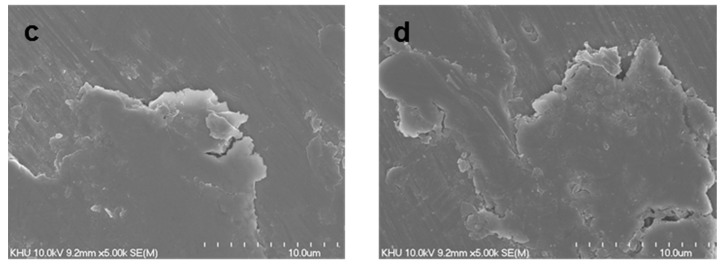
SEM images of (**a**) pristine Ti; (**b**) hBMP-2/Ti; (**c**) hGDF-5/Ti; and (**d**) hBMP-2/hGDF-5/Ti samples observed at 5000× magnification.

Figure 2 shows the surface morphologies of pristine Ti, hBMP-2/Ti, hGDF-5/Ti, and hBMP-2/hGDF-5/Ti. Compared with hBMP-2/Ti, hGDF-5/Ti, and hBMP-2/hGDF-5/Ti, a smooth surface morphology was observed on pristine Ti, indicating the formation of rougher surface by the surface modifications with hBMP-2, hGDF-5, and their combination. Some researchers reported surface modification with hBMP-2 or hGDF-5 leading to the formation of a rough Ti surface. Taken together, it was expected that the roughness of Ti samples increased with the surface modifications with hBMP-2, hGDF-5, and their combination.

### 2.3. Water Contact Angle

**Figure 3:**
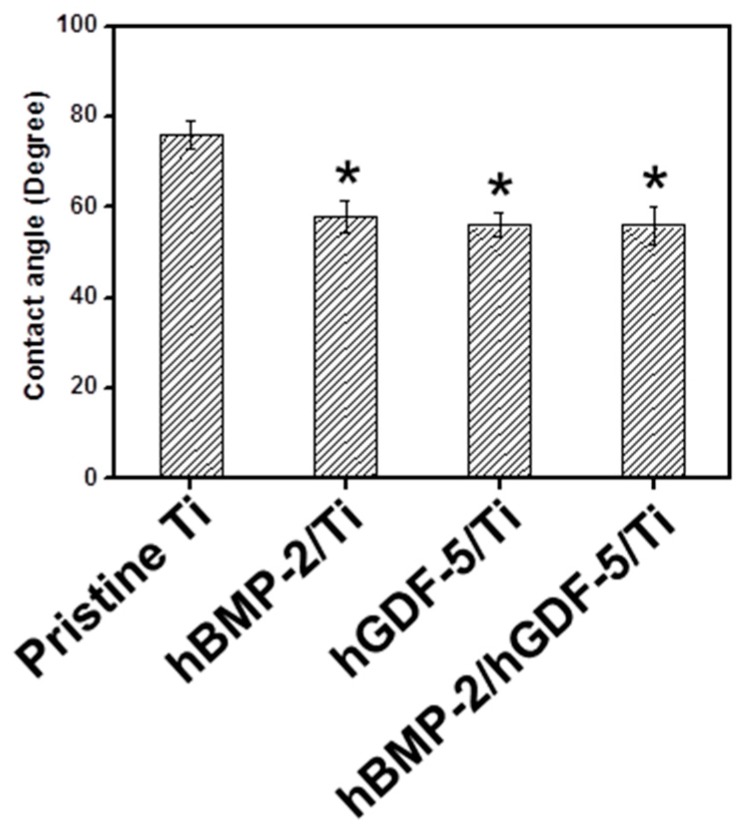
Water contact angles on pristine Ti; hBMP-2/Ti; hGDF-5/Ti, and hBMP-2/hGDF-5/Ti samples. The error bars represent mean ± SD (*n* = 3). This experiment was repeated three times (* *p* < 0.05).

The water contact angles on pristine Ti, hBMP-2/Ti, hGDF-5/Ti, and hBMP-2/hGDF-5/Ti are shown in Figure 3. Compared with pristine Ti, the surface-modified Ti samples showed decreased contact angles, indicating that the surface modifications were achieved with hBMP-2, hGDF-5, and hBMP-2/hGDF-5. The contact angles on pristine Ti, hBMP-2/Ti, hGDF-5/Ti, and hBMP-2/hGDF-5/Ti were 76, 58, 56, and 56 degrees, respectively.

### 2.4. Evaluation of Loading Efficiency and Release Behavior

**Figure 4:**
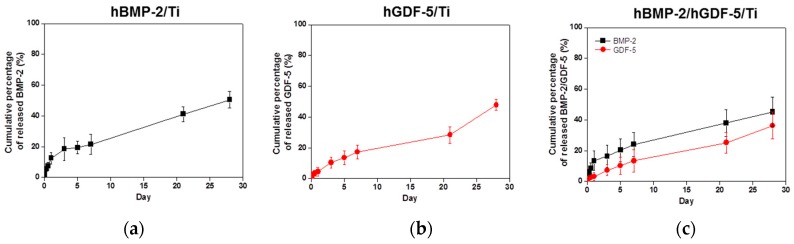
Release behavior of hBMP-2 from (**a**) hBMP-2/Ti; (**b**) hGDF-5/Ti; and (**c**) hBMP-2/hGDF-5/Ti samples in PBS (pH 7.4) monitored for 30 days. The error bars represent mean ± SD (*n* = 3). This experiment was repeated three times.

The release behavior of hBMP-2, hGDF-5, and hBMP-2/hGDF-5 on pristine Ti, hBMP-2/Ti, hGDF-5/Ti, and hBMP-2/hGDF-5/Ti was examined for 30 days (Figure 4). After an initial burst release of hBMP-2, hGDF-5, and their combination, was seen within 1 day with hBMP-2/Ti, hGDF-5/Ti, and hBMP-2/hGDF-5/Ti; thereafter, there was a sustained release of the coated growth factors in an even and measured manner. The cumulative percentages of hBMP-2, hGDF-5, and the combination, were 50.5%, 47.8%, and 45.2%/36.4%, respectively. The loading efficiencies were 76%, 73%, and 75%/73%, respectively.

### 2.5. In Vitro Cell Proliferation Rate

**Figure 5:**
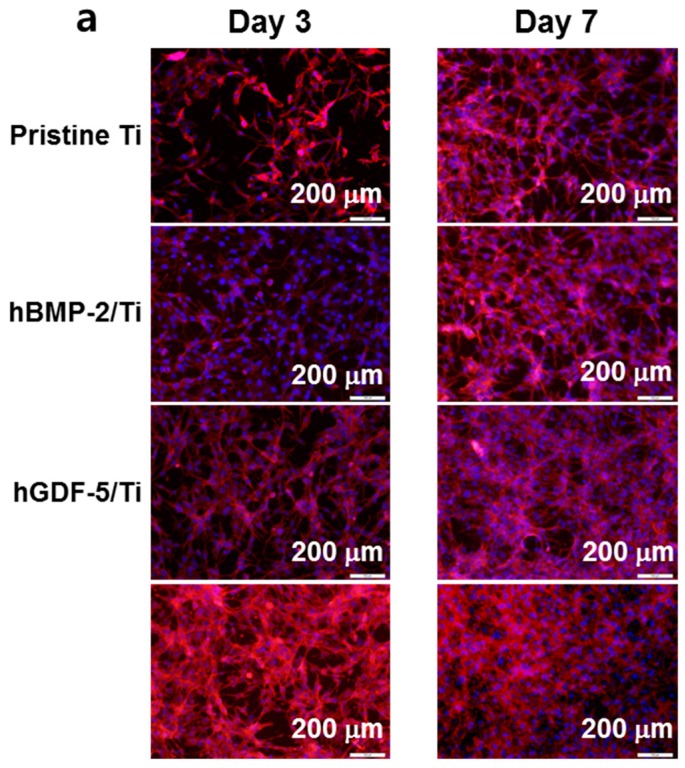

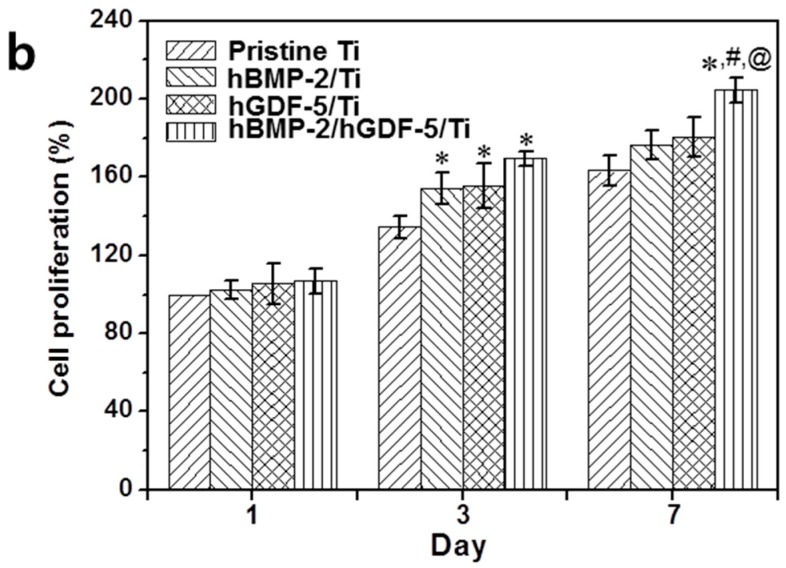
(**a**) Fluorescence microscope images (200×) and (**b**) in vitro cell proliferation rates of MC3T3-E1 cells cultured on pristine Ti, hBMP-2/Ti, hGDF-5/Ti, and hBMP-2/hGDF-5/Ti samples for 1, 3, and 7 days of culture, as compared to that of MC3T3-E1 cells cultured on culture plates (control). F-actin stain was carried out with phalloidin-Alexa Fluor 555. The error bars represent mean ± SD (*n* = 3). These experiments were repeated three times (* *p* < 0.05, ^#^ *p* < 0.05 and ^@^ *p* < 0.05). Controls of * the *p*-values were pristine Ti, hBMP-2/Ti, and hGDF-5/Ti, respectively.

The cell proliferation rate of MC3T3-E1 cells cultured on pristine Ti, hBMP-2/Ti, hGDF-5/Ti, and hBMP-2/hGDF-5/Ti for 1, 3, and 7 days was evaluated by fluorescence microscopy (Figure 5a) and CCK-8 assay (Figure 5b). A gradual increase in cell proliferation was observed for the Ti samples for seven days. The cells cultured on surface-modified Ti samples showed higher proliferation rates than those on pristine Ti throughout the culture period. In particular, cells cultured on hBMP-2/hGDF-5/Ti had a superior proliferation rate as compared with those on pristine Ti, hBMP-2/Ti, and hGDF-5/Ti. At day 7, the cell proliferation rate was 1.3-, 1.2- and 1.1-fold higher than pristine Ti, hBMP-2/Ti, and hGDF-5/Ti, respectively.

### 2.6. ALP Activity and Calcium Deposition

**Figure 6:**
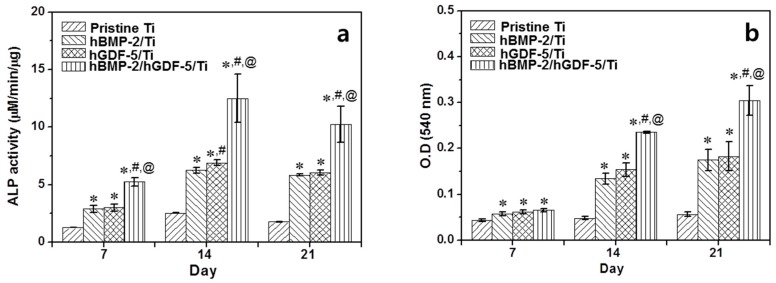
(**a**) Level of ALP activity; and (**b**) the amount of calcium deposition on pristine Ti, hBMP-2/Ti, hGDF-5/Ti, and hBMP-2/hGDF-5/Ti samples cultured with MC3T3-E1 cells for 7, 14 and 21 days, as compared to those on pristine Ti. The error bars represent mean ± SD (*n* = 3). These experiments were repeated three times (* *p* < 0.05, ^#^ *p* < 0.05 and ^@^ *p* < 0.05). Controls of the *p* values were pristine Ti, hBMP-2/Ti, and hGDF-5/Ti, respectively.

Figure 6a shows the ALP activity of MC3T3-E1 cells cultured on pristine Ti, hBMP-2/Ti, hGDF-5/Ti, and hBMP-2/hGDF-5/Ti for 7, 14 and 21 days. The ALP activity of the cells cultured on the Ti samples increased for 14 days and decreased thereafter. The cells cultured on the surface-modified Ti samples showed larger ALP activity than those on pristine Ti throughout the culture period. At day 14, the ALP activity of the cells cultured on rhBMP-2/rhGDF-5/Ti was 5.9-, 1.8- and 1.7-fold larger than cells on pristine Ti, rhBMP-2/Ti, and rhGDF-5/Ti, respectively.

The amount of calcium deposited on cell-cultured pristine Ti, hBMP-2/Ti, hGDF-5/Ti, and hBMP-2/hGDF-5/Ti for 7, 14 and 21 days is shown in Figure 6b. A gradual increase in the calcium amount was observed on the Ti samples throughout the culture period. The cells cultured on the surface-modified Ti samples showed a larger amount of calcium than those on the pristine Ti. At day 21, the amount of calcium on cell-cultured hBMP-2/hGDF-5/Ti was 5.4-, 1.7-, and 1.7-fold larger than cells on pristine Ti, hBMP-2/Ti, and hGDF-5/Ti, respectively.

### 2.7. mRNA Gene Expression

**Figure 7:**
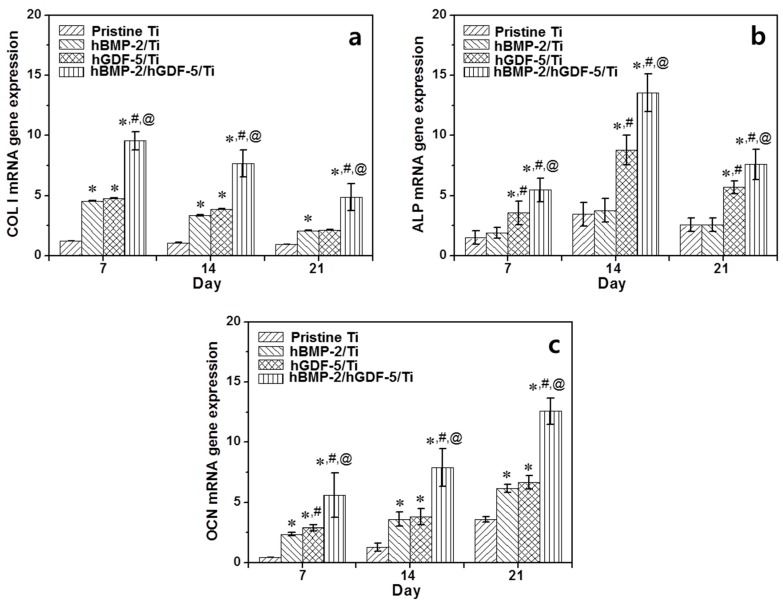
Level of (**a**) *COL 1*; (**b**) *ALP*; and (**c**) *OCN* mRNA expression for pristine Ti, hBMP-2/Ti, hGDF-5/Ti, and hBMP-2/hGDF-5/Ti samples cultured with MC3T3-E1 cells for 7, 14 and 21 days, as compared to cells on pristine Ti. The error bars represent mean ± SD (*n* = 3). These experiments were repeated three times (* *p* < 0.05, ^#^ *p* < 0.05 and ^@^ *p* < 0.05). Controls of the *p* values were pristine Ti, hBMP-2/Ti, and hGDF-5/Ti, respectively.

*COL 1*, *ALP*, and *OCN* mRNA levels were measured in MC3T3-E1 cells cultured on pristine Ti, hBMP-2/Ti, hGDF-5/Ti, and hBMP-2/hGDF-5/Ti for 7, 14, and 21 days (Figure 7). A gradual decrease in gene expression for *COL 1* on the Ti samples was observed (Figure 7a). The expression of *ALP* on the Ti samples increased for 14 days and decreased thereafter (Figure 7b). The Ti samples showed a gradual increase in *OCN* mRNA throughout the culture period (Figure 7c). At day 7, the Ti samples showed little difference in *COL 1*, *ALP* and *OCN* gene expression levels. At day 14, the genes on the surface-modified Ti samples showed larger expressions than those on pristine Ti. At day 7, the expression of the *COL 1* mRNA gene on hBMP-2/hGDF-5/Ti was 7.9-, 2.1- and 2.0-fold higher than those on pristine Ti, hBMP-2/Ti, and hGDF-5/Ti, respectively. At day 14, expression of the *ALP* mRNA gene was 3.9-, 3.6- and 1.5-fold higher than those on pristine Ti, hBMP-2/Ti, and hGDF-5/Ti, respectively. At day 21, expression of the *OCN* gene on hBMP-2/hGDF-5/Ti was 3.5-, 2.0- and 1.9-fold higher than those on pristine Ti, hBMP-2/Ti, and hGDF-5/Ti, respectively.

### 2.8. BV, BV/TV, BIC, and Removal Torque

**Figure 8:**
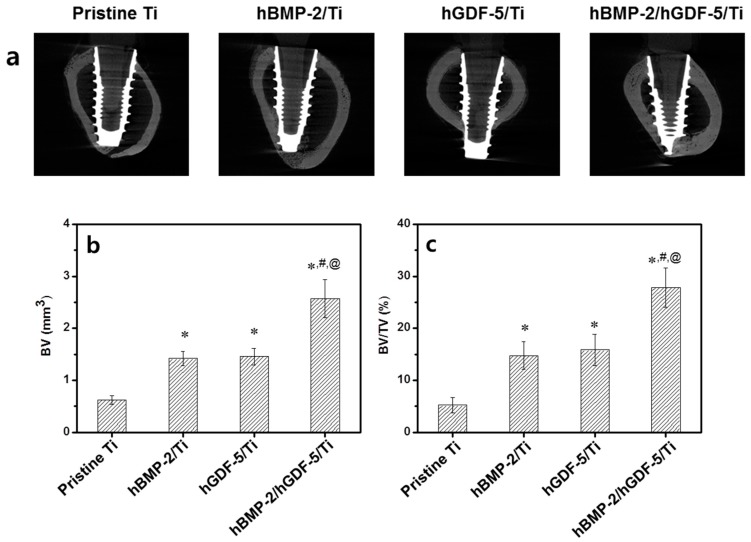

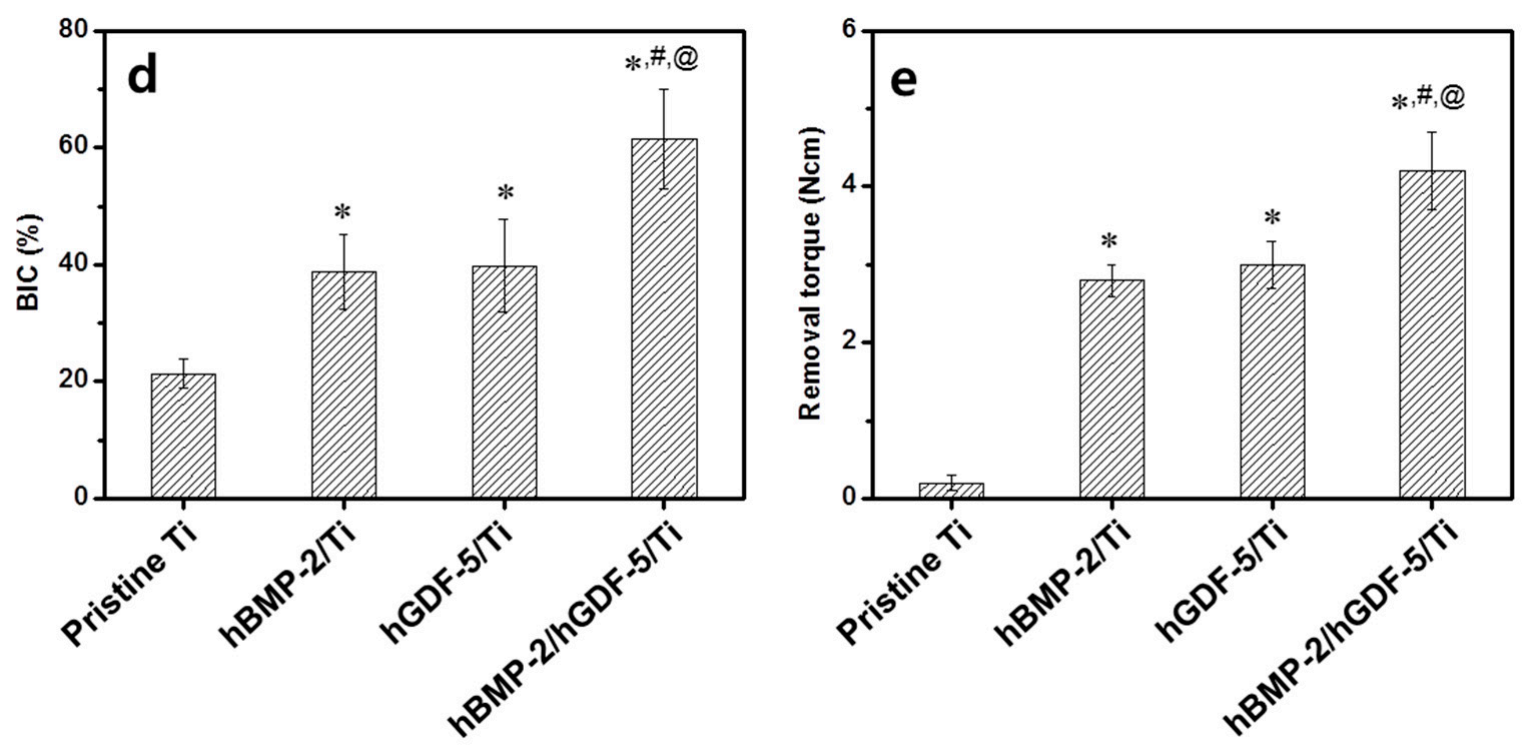
(**a**) Micro-CT images (white area: titanium screw and grey area: cortical bone); (**b**) amounts of BV; (**c**) percentages of BV/TV; (**d**) percentages of BIC; and (**e**) values of removal torque on pristine Ti, hBMP-2/Ti, hGDF-5/Ti, and hBMP-2/hGDF-5/Ti samples after four weeks of implantation. The error bars represent mean ± SD (*n* = 3). These experiments were repeated three times (* *p* < 0.05, ^#^ *p* < 0.05 and ^@^ *p* < 0.05). Controls of the *p* values were pristine Ti, hBMP-2/Ti, and hGDF-5/Ti, respectively.

Figure 8 shows BV, BV/TV, BIC, and removal torque values measured for pristine Ti, hBMP-2/Ti, hGDF-5/Ti, and hBMP-2/hGDF-5/Ti screws after four weeks of implantation. The BVs formed at the interface between the Ti implants and host bone was 0.6, 1.4, 1.5 and 2.6 mm^3^, respectively (Figure 8b). The percentage of BV/TV was 5.2%, 14.8%, 15.9% and 27.9%, respectively (Figure 8b). The percentage of BIC was 21.4%, 38.8%, 39.8% and 61.5%, respectively (Figure 8c). The value of the removal torque was 0.2, 2.8, 3.0 and 4.2 N/cm, respectively (Figure 8d). Remarkable bone formation was observed at the interface between hBMP-2/hGDF-5/Ti and host bone. This may be attributed to the synergistic effect of hBMP-2 and hGDF-5.

### 2.9. Histological Evaluations

**Figure 9:**
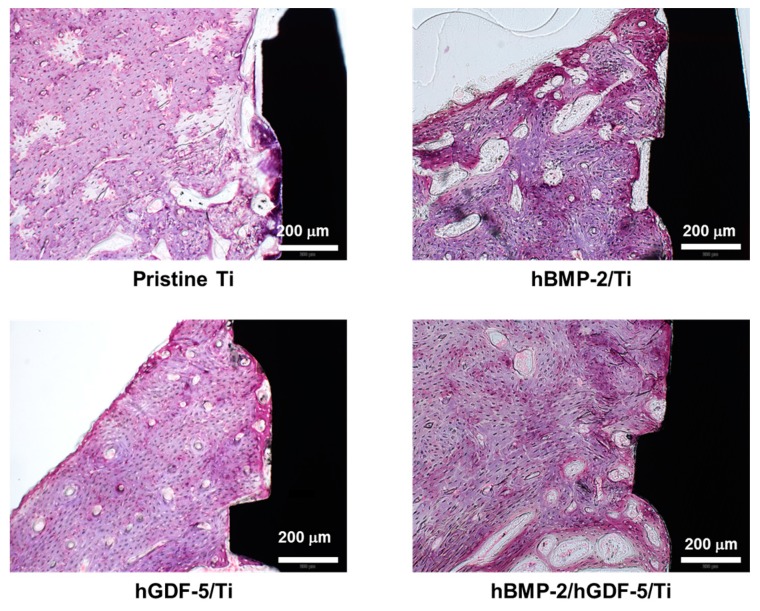
H and E stained images of the surrounding tissue around the pristine Ti, hBMP-2/Ti, hGDF-5/Ti, and hBMP-2/hGDF-5/Ti samples after implantation into femur heads for four weeks. The purple areas indicate the cortical bone of femur area. The white scale bar indicates 200 μm.

Figure 9 shows the H AND E stained images of tissues in the periphery of pristine Ti, hBMP-2/Ti, hGDF-5/Ti and hBMP-2/hGDF-5/Ti screws after four weeks of implantation. Osteoblasts, osteocytes, osteoclasts, osteons, and lacunae were observed around the tissues implanted with the Ti samples. Compared with the interface between pristine Ti and host bone, compact bone formation was observed at the interfaces between the surface-modified Ti samples and host bone. In particular, hBMP-2/hGDF-5/Ti showed enhanced bone formation at the interface with host bone, indicating activation of bone remodeling. In addition, lamella bone and bone ingrowth toward the interface were found at the tissue around hBMP-2/hGDF-5/Ti. Both empty and osteocyte-occupied lacunae were observed on the tissue between the Ti samples and host bone. These results indicated that the surface-modified Ti samples enhanced bone formation at the interface between the Ti samples and the host bone. The hBMP-2/hGDF-5/Ti especially showed remarkable bone formation at the interface.

## 3. Discussion

The surface treatment of Ti implants affects their wettability, morphology, and release behavior of biomolecules [3]. These parameters play important roles in obtaining a successful implantation [3]. Thus far, most physical or chemical surface modifications developed for refining these parameters [16,17,18,19] have been unsuccessful in achieving the improvements required. However, biological surface modification with the BMP family of proteins was recently suggested as a new tool for advancing these purposes [3,5,6,12].

Among the BMP family, hBMP-2 and hGDF-5 were chosen for this study due to their ability on osteogenic differentiation of mesenchymal stem cells. However, in spite of their merits, several problems remain, including their high cost, the required high doses (from several micrograms up to milligrams), and their relatively short half-life [3,5,6,12]. To address their short half-life, heparin, a glycosaminoglycan, was used as it inhibits the degradation of BMP-2 by decreased the binding of noggin, a potent inhibitor of BMP-2 as its mRNA is induced by BMP-2 [15]. Noggin uses the heparin binding site of BMP-2 to bind to BMP-2, and heparin competitively inhibits the binding of noggin to osteoblasts, resulting in a prolonged BMP-2 half-life. Zhao and colleagues reported that the half-life of BMP-2 was prolonged nearly 20-fold by adding heparin [20]. Additionally, Chun and co-workers reported that heparin/BMP-2-coated Ti improved cell proliferation and differentiation of osteoblasts [3]. GDF-5 is also known to bind heparin due to its heparin-binding site [15,21]. Lee and colleagues reported the effect of GDF-5-coated zirconia on enhancement of bone formation [10]. Taken together, surface modification with the combination of BMP-2 and GDF-5 on Ti may enhance bone formation at the interface between the implant and the host bone, thereby inducing strong osseointegration with the host bone.

In the present study, we designed and prepared hBMP-2/hGDF-5-coated Ti implants of disc and screw types using heparin, and their efficacy on enhancing bone formation and osseointegration was evaluated in vivo as compared to pristine Ti, hBMP-2/Ti, and hGDF-5/Ti. The chemical composition analysis of the Ti samples indicated their successful surface modification (Figure 1).

Surface morphology and wettability of Ti implants are important factors that affect the initial osseointegration for a successful implantation [22]. The response of cells on Ti implants is modulated by their roughness and wettability [22]. The combination of micro- and nanostructures, along with the hydrophilicity of the metal surfaces, has been proven to enhance osteoblast differentiation and local production in vitro and osseointegration in vivo [3,23]. The modifications with hBMP-2, hGDF-5, or hBMP-2/hGDF-5 produced more rough and hydrophilic surface than with pristine Ti (Figure 2 and Figure 3).

Controlled release of hBMP-2 or hGDF-5 on Ti implants allows for enhanced bone formation, leading to stable initial osseointegration. Some researchers introduced the surface modifications of Ti implants for their controlled releases [3,5,12]. Among the surface modifications, heparin was used for the controlled release of hBMP-2 or hGDF-5 through their specific interaction [3,5,12]. As expected, our results also indicated that hBMP-2, hGDF-5 and the double layer-coated Ti implants with heparin released them in a sustained manner (Figure 4).

Osteogenic differentiation of mesenchymal stem cells on Ti implants includes the three steps of proliferation, bone maturation and bone mineralization [24], and the osteogenic differentiation is related to stable osseointegration of the implants [24]. The osteogenic differentiation starts from adhesion and proliferation of several cell types, such as mesenchymal stem cells, pre-osteoblasts, and osteoblasts [24]. Here, we suggested that the surface modification with hBMP-2, hGDF-5 or their combination enhances the proliferation of MC3T3-E1 cells, ALP activity and calcium deposition (Figure 5 and Figure 6). *COL 1*, *ALP*, and *OCN* are representative bone-relating mRNAs for proliferation, bone maturation and bone mineralization steps, respectively [25,26,27]. In general, the mRNA expression of *COL 1* decreases throughout the culture period [25,26,27], and that for *ALP* increases for up to two weeks and decreases thereafter [25,26]. *OCN* message increases throughout the culture period [25,26,27]. Our in vitro data also showed the same pattern for the mRNA changes (Figure 7) [3].

In this study, micro CT analysis was carried out to evaluate the efficacy of the surface modifications on enhancement of bone formation and osseointegration [3,27]. H and E stain was also carried out for evaluation of the effect of surface modifications on behavior of bone-related cells, including osteoblasts, osteocytes, and osteoclasts in the implant-bearing tissues [3]. Herein, we postulated that the double-layered modification with rhBMP-2 and rhGDF-5 on the surface of Ti implants can enhance bone formation and osseointegration at the interface between the surface-modified implants and host bone due to the osteogenic activity of hBMP-2 and hGDF-5. Some groups have also reported the effect of hBMP-2 or hGDF-5-coated Ti implants on the enhancement of bone formation in vivo [3,5,12]. As expected, active bone remodeling happens at the interface between hBMP-2/hGDF-5/Ti and host bone through a process of ossification by osteoblasts and bone resorption by osteoclasts (Figure 8 and Figure 9). Consequently, the results indicated that there was a synergistic effect of hBMP-2 and hGDF-5 with hBMP-2/hGDF-5/Ti, contributing to enhanced bone formation and osseointegration.

## 4. Experimental Section

### 4.1. Materials

Titanium (Ti), in the form of disc and screw types with a smooth surface (diameter 8 mm, height: 1 mm for the disc type; and φ 3.5 mm × 8.5 mm for the screw type), were kindly provided by DIO Co. (Busan, Korea). Dexamethasone acetate (DEX), β-glycero phosphate disodium salt hydrate, ascorbic acid and dexamethasone were purchased from Sigma-Aldrich (St. Louis, MO, USA). Heparin (12,000–15,000 g/mol) was purchased from Acros Organics/Thermo Fisher (Waltham, MA, USA). Dulbecco’s Modified Eagle Medium (DMEM), fetal bovine serum (FBS), phosphate-buffered saline (PBS), and penicillin-streptomycin (PS) solutions were purchased from Gibco BRL (Gaithersburg, MD, USA). 4-(4,6-Dimethoxy-1,3,5-triazin-2-yl)-4-methylmorpholinium chloride (DMT-MM) was purchased from Wako (Osaka, Japan). Human bone morphogenetic protein-2 (hBMP-2), human growth and differentiation factor-2 (hGDF-5), and their ELISA kits were purchased from Peprotech Inc. (Rocky Hill, NJ, USA). MC3T3-E1, an osteoblast precursor cell line, was supplied from the Korean Cell Line Bank (Seoul, Korea). All organic solvents were used as received without any additional purification.

### 4.2. Preparation of hBMP-2/hGDF-5-Coated Ti Surface (hBMP-2/hGDF-5/Ti)

**Figure 10:**
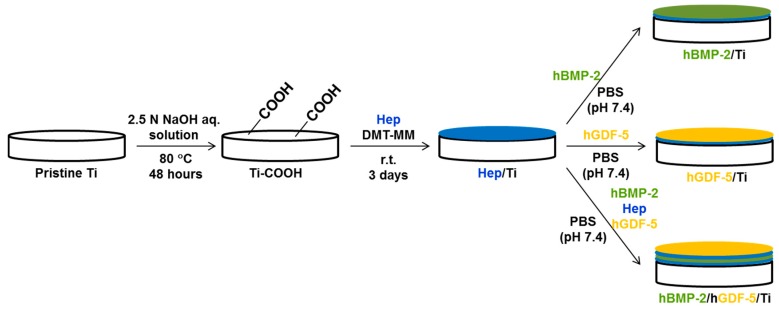
Schematic for the preparation of pristine Ti; BMP-2/Ti; GDF-5/Ti; and BMP-2/GDF-5/Ti discs (diameter: 8 mm; height: 1 mm) and screws (φ 3.5 mm × 8.5 mm). The two types of Ti samples were surface-modified by the same process.

A schematic of the preparation of hBMP-2/Ti, hGDF-5/Ti, and hBMP-2/hGDF-5/Ti is shown in Figure 10. Briefly, pristine Ti disc and screw types were added to 2.5 N of aqueous alkali NaOH solution (500 mL) and continuously stirred at 80 °C for 48 h. After ultra-sonication for five minutes in distilled water, the discs were washed with distilled water three times and dried with nitrogen gas (Ti–COOH). Ti–COOH was activated with DMT-MM (1 mmol, 27 mg) at room temperature for one hour. After addition of heparin (1 mmol, 10 mg) to a mixture of the activated Ti-COOH in distilled water, the mixture was reacted at room temperature (r.t.) for three days. After washing the reactant three times with distilled water, the discs were dried with nitrogen gas and stored in desiccator filled with nitrogen gas before use (Hep/Ti). hBMP-2 (50 ng), heparin (10 mg), and hGDF-5 (50 ng) were coated on Hep/Ti in PBS (40 mL, pH 7.4), in that order, and the procedure was carried out at 4 °C for 24 h. After washing the discs with distilled water three times, the resultant Ti metal fixtures were dried with nitrogen gas and stored in a refrigerator (−20 °C) before use. For comparative studies, rhBMP-2/Ti and rhGDF-5/Ti metal implements were prepared according to the referred protocol. The amount of hBMP-2 and hGDF-5 used for both was 50 ng.

### 4.3. Pristine Ti, hBMP-2/Ti, hGDF-5/Ti, and hBMP-2/hGDF-5/Ti of Disc and Screw Types

The Ti disc samples were analyzed by X-ray photoelectron spectroscopy (XPS), scanning electron microscopy (SEM), contact angle measurement, and release test. They were tested for their in vitro cell proliferation, alkaline phosphatase (ALP) activity, and calcium deposition properties. The Ti screw type samples were used for in vivo animal tests.

### 4.4. XPS

The surface element compositions of pristine Ti, hBMP-2/Ti, hGDF-5/Ti, and hBMP-2/hGDF-5/Ti were examined using a K-Alpha XPS system (Thermo Electron Corp, Thermo Fisher, Waltham, MA, USA). The source was a 10 kV, ~30 mA monochromated X-ray beam (photoelectron energy = 1486.6 eV) obtained from an aluminum anode. The wide scan was recorded from 100 and 800 eV under a grazing angle of 90° in a high vacuum (<3.1 × 10^−9^ Torr).

### 4.5. SEM

Pristine Ti, hBMP-2/Ti, hGDF-5/Ti, and hBMP-2/hGDF-5/Ti on metal mounts were gold-coated using an ion sputter-coater (Eiko IB-3, Eiko Engineering Co., Ltd., Hitachinaka, Japan). The surface morphologies were examined by SEM (S-2300, Hitachi Corp., Hitachi, Japan) at 15 kV, as observed at 5000× magnification.

### 4.6. Contact Angle Measurement

Water contact angles on pristine Ti, hBMP-2/Ti, hGDF-5/Ti, and hBMP-2/hGDF-5/Ti were measured using an Erma G-1 contact angle meter (Tokyo, Japan) on a droplet of 20 μL of water on the surface of the Ti samples.

### 4.7. Loading Efficiency and In Vitro Release Test of hBMP-2, hGDF-5, or hBMP-2/hGDF-5

After the coating process shown in Section 2.2, 100 μL of the remaining solution was analyzed by a microplate reader (Bio-Rad, Hercules, CA, USA) using an enzyme-linked immunosorbent assay (ELISA) for the calculation of loading efficiency. The surface-modified Ti samples, hBMP-2/Ti, hGDF-5/Ti, and hBMP-2/hGDF-5/Ti were immersed in 3 mL of PBS (pH 7.4) and incubated at 37 °C at 100 rpm. At each predetermined time interval (1, 6, and 12 h, and 1, 3, 5, 7, 14, 21 and 28 days), 3 mL of the supernatants were collected and an equal volume of fresh PBS was added to the sample. The collected supernatants were measured by a microplate reader using ELISA for examining the amount of rhBMP-2 and rhGDF-5 coated onto Ti.

### 4.8. Culture of MC3T3-E1 Cell Line

MC3T3-E1 cells were cultured in 48-well plates with 200 μL of DMEM containing 10% FBS and 1% PS in a humidified incubator maintained at 37 °C and supplemented with 5% CO_2_. The medium was changed every three days during culture.

### 4.9. In Vitro Cell Proliferation Rate

MC3T3-E1 cells (5 × 10^3^ cells/well) were seeded on pristine Ti, hBMP-2/Ti, hGDF-5/Ti, and hBMP-2/hGDF-5/Ti placed in 48-well culture plates with 200 μL of DMEM containing 10% FBS and 1% PS and incubated for three days. At each of the predetermined time intervals, the nuclei and cytoskeletons of the cells cultured on the samples were stained by 4′-6-diamidino-2-phenylindole (DAPI) and F-actin method using rhodamine phalloidin (Invitrogen, Waltham, MA, USA), as observed by fluorescence microscope (OLYMPUS DP2-BSW, NY, USA) at 200×. Additionally, the samples were collected and the CCK-8 assay was performed to evaluate cell proliferation according to the manufacturer’s instructions.

### 4.10. Alkaline Phosphatase (ALP) Activity Assay

MC3T3-E1 cells (1 × 10^5^ cells/well) were seeded on pristine Ti, hBMP-2/Ti, hGDF-5/Ti, and hBMP-2/hGDF-5/Ti in 48-well culture plates with osteogenic media containing 10% FBS, 1% PS, 10 mM β-glycero phosphate disodium salt hydrate, 300 μM ascorbic acid, and 0.1 μM dexamethasone, and incubated for 7, 14 and 21 days. At each predetermined time interval, the samples were washed with PBS (pH 7.4) and radioimmunoprecipitation assay buffer (RIPA buffer, 50 mM Tri-HCl pH 7.4, 150 mM NaCl, 0.25% deoxycholic acid, 1% Nonidet P-40 (NP-40), and 1 mM ethylenediaminetetraacetic acid (EDTA) containing protease inhibitor cocktail (Roche, Mannheim, Germany) was added. The cells were lysed in RIPA buffer for 20 min on ice and the lysates were centrifuged at 4 °C for 10 min to remove cell debris. The supernatants were incubated with *p*-nitrophenyl phosphate (PNPP), and the reaction with PNPP was terminated by adding 50 μL of 1 N NaOH. ALP activity was determined by measuring the conversion of PNPP to *p*-nitrophenol at 410 nm, as determined by a microplate reader.

### 4.11. Assay of Calcium Deposition

MC3T3-E1 cells (1 × 10^5^ cells/well) were cultured onto pristine Ti, hBMP-2/Ti, hGDF-5/Ti, and hBMP-2/hGDF-5/Ti discs for 7, 14 and 21 days. The discs rinsed at each of the culture times were fixed with 3.7% formaldehyde for 20 min and rinsed again. For alizarin S-red stain, the discs treated with 40 mM of the solution were incubated at 37 °C for one hour under 50% CO_2_. Afterward, the extracted discs were rinsed with distilled water three times. 1-hexadecylpyridinium chloride (10%) was used for desorption of the stained cells, and measured at 540 nm using a microplate reader for quantifying the amount of calcium deposited onto the stained cells.

### 4.12. Analysis of Real-Time Polymerase Chain Reaction (qPCR)

RNeasy Plus Mini Kit (Qiagen, Germantown, MD, USA) was used for the isolation of total RNA on the MC3T3-E1 cultured Ti discs for 7, 14 and 21 days. After extracting 1 μg of total RNA from the discs, it was transcribed into cDNA with AccuPower CycleScript RT Premix (Bioneer, Daejeon, Korea). An AccuPower PCR PreMix (Bioneer, Daejeon, Korea) was used for qPCR amplifications. An iQ SYBR Green supermix (Bio-Rad, Hercules, CA, USA) was used for the detection of qPCR products. The values of the threshold cycle were determined by using a comparative CT method. At each analysis time, the fold change of the control condition was set as one fold. Table 1 shows the primers used for this study. The qPCR amplifications were carried out under a cycle of 10 s at 95 °C, 30 s at 57–62 °C (*COL 1*: 60 °C, *ALP*: 61 °C, *OCN*: 57 °C), and 30 s at 72 °C for 45 cycles after the initial denaturation step for 10 min at 95 °C [15].

### 4.13. In Vivo Animal Test

Ten white New Zealand rabbits (2.5–3.5 kg, *n* = 10, D.Y. Biotech., Seoul, Korea) were used for in vivo animal testing [3]. Ketara and Rompun were used for anesthetizing the animals. They were disinfected with Betadine, followed by shaving of their femur and proximal tibia. After the injection of a Lignospan standard (0.5 mL) into the subcutaneous tissues, the sites were cut with a surgical scalpel. Five screws for micro-CT and histological analyses, and five screws for removal torque value measurement per each Ti sample, were implanted into the femur and the proximal tibia, respectively. Periosteum, fascia, and skin were sutured with Ethicon and Ethicon Ethilon, respectively. Cefamandole was used for three days for preventing further complications after the surgery. After four weeks, the tissues around the implanted sites were harvested after sacrificing the animals with KCl. The sites were evaluated by micro-CT and removal torque measurements.

### 4.14. Scan of Micro-CT Images

Micro-CT (SkyScan 1076, Bruker, Brussels, Belgium) was used for the examination of bone volume (BV), bone volume/tissue volume (BV/TV), and bone-implant contact (BIC) between the Ti samples and host bone. Conditions of the measurement are as follows: X-ray source voltage of 100 kV, X-ray source current of 100 μA, 0.6 degree angular increments over 360 degrees and exposure for 460 ms. A distance of 1.4 mm from longitudinal axis of the samples was selected as region of interest (ROI). Skyscan CT-analyzer software (Bruker) was used for the analysis of the scanned images. For calibration of the acquired images, the CT number of air and water were set as −1000 HU and 0 HU, respectively.

### 4.15. Measurement of Removal Torque

A computerized material testing machine (100 R, Admet, Norwood, MA, USA) was used for the measurement of removal torque between the Ti samples and host bone. After fixing of the Ti sample-implanted tissues, the screw was turned with the machine at a speed of 0.4 degrees/s. The maximum toque was selected for removal torque value of the Ti samples.

### 4.16. Histological Analyses

The Ti sample-implanted tissues were fixed with 10% formalin solution and dehydrated with various grades of ethanol (80%, 90%, and 100%). After embedding the tissues in acrylic resin, the mixtures were polymerized by a light polymerization unit (EXAKT 520, EXAKT Technologies Inc., Oklahoma, OK, USA). The center of each resin block was cut with a diamond band cutting system (EXAKT 300CP, EXAKT Technologies Inc., Oklahoma, OK, USA) and ground with a micro-grinding system (EXAKT 400CS, EXAKT Technologies Inc.) to make slides with a thickness of 40 μm. The slides were stained with hematoxylin and eosin (H AND E) and observed by fluorescence microscopy (AX70, TR-62A02, Olympus, Tokyo, Japan).

### 4.17. Statistical Analysis

One-way analysis of variance (ANOVA) using SPSS software (SPSS Inc., Chicago, IL, USA) was used for performing statistical analysis, and expressed as the mean ± standard deviation. Values of * *p* < 0.05, ^#^ *p* < 0.05, and ^@^ *p* < 0.05 were considered statistically significant. MedCalc statistical software (MedCalc Software bvba, Ostend, Belgium) using alpha (*p* = 0.05) and power (1 − β = 0.8) was used for the calculation of the number of rabbits used for this study. 

### 4.18. Institutional Review Board

The Institutional Animal Care and Use Committee (IACUC) of the Kyung Hee University Hospital at Gangdong approved in vivo animal testing for this study (No. KHNMC AP 2016-008).

## 5. Conclusions

Ti metal surfaces of disc and screw types were modified by a combination of hBMP-2 and hGDF-5 (hBMP-2/hGDF-5/Ti), and their efficacy for enhancement of bone formation and osseointegration was evaluated in vivo, as compared to pristine Ti, hBMP-2/Ti, and hGDF-5/Ti. Together with their modified surface morphology and wettability, the controlled release of hBMP-2 and/or hGDF-5 from the coated surfaces led to enhanced bone formation and osseointegration. In conclusion, hBMP-2/hGDF-5/Ti modification has the potential for clinical use for improved dental or orthopedic implants.

## Figures and Tables

**Table 1:** Accession numbers and primers of type I collage (*COL 1*), *ALP*, osteocalcin (*OCN*), and glyceraldehyde 3-phosphate dehydrogenase (*GAPDH*) mRNA genes used for this study.

Genes	Accession Number	Primers	
Type I collagen (*COL 1*)	XM005257059.3	Sense	5′-GTGAGACAGGCGAACAAG-3′
		Antisense	5′-CAGGAGAACCAGGAGGAC-3′
*ALP*	XM017001582.1	Sense	5′-CCAGCAGGTTTCTCTCTTGG-3′
		Antisense	5′-GACTGTAGGGACGATTGGA-3′
osteocalcin (*OCN*)	NM172209.2	Sense	5′-ATGAGGACCCTCTCTCTGCT-3′
		Antisense	5′-CCGTAGATGCGTTTGTAGGC-3′
*GAPDH*	NM001289746.1	Sense	5′-ACTTTGTCAAGCTCATTTCC-3′
		Antisense	5′-TGCAGCGAACTTTATTGATG-3′
